# On the self-regulation of sport practice: Moving the narrative from theory and assessment toward practice

**DOI:** 10.3389/fpsyg.2023.1089110

**Published:** 2023-03-28

**Authors:** Bradley W. Young, Stuart G. Wilson, Sharleen Hoar, Lisa Bain, Malgorzata Siekańska, Joseph Baker

**Affiliations:** ^1^School of Human Kinetics, University of Ottawa, Ottawa, ON, Canada; ^2^Canadian Sport Institute Pacific, Victoria, BC, Canada; ^3^Department of Psychology, University of Physical Education in Krakow, Kraków, Poland; ^4^School of Kinesiology and Health Science, York University, Toronto, ON, Canada

**Keywords:** self-regulated learning, applied survey use, translating theory-to-practice, psychology of practice, deliberate practice

## Abstract

This paper reviews theoretical developments specific to applied research around the “psychology of practice” in skill acquisition settings, which we argue is under-considered in applied sport psychology. Centered upon the *Self-Regulation of Sport Practice Survey (SRSP)*, we explain how self-regulated learning conceptually underpins this survey and review recent data supporting its empirical validation for gauging athletes’ psychological processes in relation to sport practice. This paper alternates between a review of applied research on self-regulated sport practice and new data analyses to: (a) show how scores on the SRSP combine to determine an expert practice advantage and (b) illustrate the large scope of self-organized or athlete-led time to which SRSP processes may apply. At this stage, the SRSP has been established as a reliable and valid tool in the empirical, theoretical domain. In order to move the narrative from theory and assessment toward applied practice, we present evidence to propose that it has relevance as a dialogue tool for fostering meaningful discussions between athletes and sport psychology consultants. We review initial case study insights on how the SRSP could be located in consultation in professional practice, propose initial considerations for its practical use and invite practitioners to examine its utility in applied settings.

## Introduction

Sport psychology has long focused on those strategies, skills, and attributes that allow athletes to perform better than ever in competition. To create value for sport psychology services, at intake, many sport psychology consultants (SPCs) ask athletes to reflect on the percentage of time they spend mentally training relative to physically training. Commonly, the resulting incongruence—largely in favor of physical training—sparks athletes’ interest in pursuing sport psychology training (Weinberg and Gould, 2019). We contend, however, that there is another aspect on incongruence that is insufficiently broached when it comes to sport psychology. Specifically, we hypothesize that a “psychology of sport practice,” dedicated to psychologically enhancing the context where athletes spend the vast majority of their sport-related time, is underappreciated. The considerable time athletes spend practicing raises questions about the value of psychological skills for optimizing time in practice. Given the primacy of practice in how athletes spend their time, why does the content of mental skills application focus predominantly on competitive readiness, or performance-enhancement centered on competitive events? If scholars hold that high-quality practice, accrued over years of development, is the critical determinant of making a national team or Olympics (Baker and Young, 2014; Côté et al., 2014; Baker et al., 2020), is there a way to activate mental skills to enhance the psychology of practice?

These are questions of *context* that have underpinned the interests of our research team, comprising members with applied and theoretically grounded perspectives. Of course, performance-enhancement facets pertaining to competitive readiness need to be practiced. This is undeniable and represents the raison d’être for many consultants. However, we contend that applied strategies/skills have infrequently been dedicated to psychology *within* sport training. The focus of sport psychology has not been on applying skills to explicitly enhance athletes’ process of preparation (i.e., practice). Practice has been addressed by SPCs in so much as it is an antecedent, or indirect determinant, of competitive readiness, especially with respect to confidence and anxiety ahead of events. The motivation to practice is a common applied topic with athletes, but practice behaviors are the outcome of such discussion—cognitions and regulatory strategies *during practice* are not the targets of discussion *per se*. Moreover, the field of sport psychology’s increasing divergence from prior traditions of motor learning (which focused on skill acquisition and practice design), its increasing theoretical focus on social psychology orientations, and the marrying of dominant applied sport psychology narratives with performance enhancement but not necessarily practice enhancement (Janelle, 2001), has meant less space devoted to practice (cf., Thomas et al., 1999). Even those practitioners who work with athletes to prepare them for training do so without a strong evidentiary foundation, for example, by equally assessing mental skills for both competition and practice. Altogether, we submit that narratives on the psychology of practice are under-represented in popular sport psychology consultation and there needs to be renewed attention on *how* athletes regulate thoughts, metacognitions, motivations and feelings *during practice*. Thus, the current paper is a review of theoretical developments around the psychology of practice, with an aim of proposing the novel and specific implications these developments have for applied research in sport settings.

Pondering the value of a “psychology of sport practice” may be seen as an exercise in contemplating “margins of gain” in high performance sport. A key question to ask is “do athletes stand to gain increasing margins of performance enhancement if they begin to explicitly attend to how they self-regulate their learning in training/practice?”. Yet, much literature has been affixed to the training of the psyche in the immediate lead-up to, and during competitions, or what Oglesby (2016) described as being highly focused on the “now” of performance and fixated on “self-regulation in the moment of performance” (p. 537). The exception is the Test of Performance Strategies (TOPS) (Thomas et al., 1999) survey, which addresses competition *and* practice. Its purpose is to assess use of identifiable psychological mental skills, for which it does a good job in relation to practice. Yet this survey assumes an entry point to consultancy where athletes get assessed on their use of an inventory of traditional skills (e.g., imagery, goal setting, self-talk) (Gould et al., 2002; Frey et al., 2003; Hardy et al., 2010). Asking athletes to judge their awareness of skill use is not the same as asking them to judge their awareness of metacognitions and motivations (and how they regulate such aspects) around practice. The latter approach initiates a comprehensive examination of an athlete’s foundational self-awareness processes and thus may be a more fruitful and generative portal to a client-consultant discussion in the practice context, which may later be followed by mental training skills assessment and application of methods (Vealey, 2007).

The aim of this paper is to encourage practitioners to consider the importance of addressing the psychology of practice. We do so by discussing the evolution of the *Self-Regulation of Sport Practice Survey* (the *SRSP* survey), a tool specifically designed to assess, emphasize, and enhance this topic. This survey has demonstrated reliability and validity of assessment within competitive athlete cohorts and has been featured in narratives around athlete development. Still, one of the original intents in creating this instrument was practical validity and value to practitioners, an area where it has yet to be examined extensively. We propose that the survey is sufficiently ready for critical examination in the applied world. (In making this case, we have integrated some sections in this paper with details on original data analyses not previously reported. These original data were derived from research in keeping with ethics approval from the University of Ottawa Research Ethics and Integrity Board: H-08-21-7176, including participant informed consent.) We do not pretend that the narrative regarding the SRSP survey is the only, the best, or the most complete narrative on the psychology of practice. That said, the aim of this paper is to invite practitioners interested in the translation of theory to practice to consider the potential novelty of this SRSP survey in applied settings, and prospectively in dialogue around consulting practice.

The SRSP has 31 items that ask athletes about the psychological processes (not mental skills) they use to optimize their sport practice (Supplementary Appendix A). It prompts them to consider their training, and the types of practice tasks they find really challenging, that they feel are important for their sport development, and that are not necessarily enjoyable to do. This prompt is informed by the construct of deliberate practice (Ericsson, 2020), the notion that not all practice activities are equal, and that engagement of conscious processes during critical activities brings forth the greatest skill acquisition gains (Baker et al., 2020; Young et al., 2021). After the prompt, athletes respond to statements relating to five different processes (subscales) on a Likert-scale.

Three of these subscales are metacognitive in that they assess athletes’ thinking about their thinking, during practice: *Planning*, relates to cognitions about goal-oriented approaches to practice tasks, deciding on approaches to a task, and task analysis; *Checking*, involves tracking what one is doing during practice tasks and checking back on procedures when done; *Evaluating-Reflecting* describes post-practice cognitions in which one evaluates their practice performance to elicit information on strengths and weaknesses, improvement, and insights that can be applied for future learning efforts. Two subscales are motivational: *Effort*, pertains to beliefs about one’s capability to recruit personal effort and concentration during practice tasks and perseverance in the face of hard tasks; *Self-Efficacy for Challenge* refers to beliefs about one’s capability to successfully perform practice tasks in unforeseen/difficult situations, involving coping and resourcefulness. In addition to individual subscale scores, all five scores can be averaged to derive a composite/overall score, which indicates the degree of engagement of *self-regulated learning* an athlete reports towards their practice tasks.

## Social cognitive foundations of self-regulated learning

The term “self-regulation” is ubiquitous in behavioral sciences, particularly in sport psychology. Few sport psychology scholars, however, distinguish between self-regulation in relation to competition or practice (cf., Crews, 1993; Thomas et al., 1999; McCardle et al., 2019). The use of the term *self-regulated learning* (SRL) links to an established scholarly lineage in educational psychology, in which SRL has been associated with better academic performance, achievement striving, and study habits (Zimmerman and Schunk, 2001; Nota et al., 2004). Self-regulated learners demonstrate an awareness of personal strengths and weaknesses, of resources they can apply to meet complex demands of learning situations, and how to manage their behaviors in various drills to optimize learning (Winne and Perry, 2000).

SRL concerns how people are meta-cognitively, motivationally, and behaviorally active in their own learning process (Zimmerman, 1998). Zimmerman and Schunk (2001) conceptualized SRL as being enacted in temporal phases (i.e., forethought, performance, self-reflection) and located key psychological processes within those phases as part of a SRL cycle. The subscales of the SRSP survey derive from key psychological processes in this cyclical model. As early as 1998, Zimmerman (1998) extrapolated his perspectives on developing students’ personal agency in study habits through SRL to people aspiring to become sport experts. Further work in experimentally-controlled environments linked SRL processes with enhanced practice habits in elite team sport players compared to less-elite players (e.g., Cleary and Zimmerman, 2001). The subscales in the SRSP consider how athletes plan, appraise, monitor, and react during practice, which is an essential difference from the TOPS survey (Thomas et al., 1999), which is based in the tradition of identifying mental skills related to peak performance (Williams and Krane, 2001).

The development of the SRSP is associated with a notable lineage of research. It evolved from Toering et al.’s (2009; 2012) work, which was instrumental in moving the concept of SRL into the purview of sport researchers, with a focus on survey-based methods. Their works borrowed on Zimmerman’s conceptualization, further intonated by Ertmer and Newby’s (1996) notion of a reflective expert learner. They pursued behavioral observational strategies, coach ratings, and tests of construct validity with measurement models to create the Self-Regulated Learning – Self-Report Scale (SRL-SRS) (Toering et al., 2012). This survey borrowed scales from education and other non-sport domains, including generalized self-efficacy measures. Studies using the SRL-SRS showed consistent differences between expert and less-expert athletic groups on the reflection subscale (Toering et al., 2009; Jonker et al., 2010), and associated self-reflection with improved skill development (te Wierike et al., 2018).

The SRL-SRS was a *dispositional* instrument “intended to measure self-regulation as a relatively stable attribute in multiple learning domains” (Toering et al., 2012, p. 25), such as sport and school. The survey became notable in examining aptitudes of school-aged athletes (often in sport academies) and interrogating whether the same SRL competencies would apply equally in the sport *and* school realms (e.g., Jonker et al., 2011; Wilson et al., 2021a). For example, authors of a Chinese SRL-SRS version noted its value regarding SRL in classrooms, physical education and sport, with implications for performance and health behaviors (Pitkethly and Lau, 2016), though little consideration was given to sport practice.

As the SRL-SRS was being developed, Young and Starkes (2006a,b) were investigating the nature of self-regulated sport practice and the role of self-monitoring for enhancing practice behaviors (Young et al., 2009). Young and Medic (2008) articulated the relevance of Zimmerman’s SRL cycle for developing athletes’ agency during self-practice, advancing the idea that skill acquisition depends on an athlete engaging in self-enhancing cycles of learning over repeated trials, across time. These works, along with work by Cleary and Zimmerman (2001) suggested that SRL was very much context-specific and tailored by athletes to the tasks they encountered in sport practice. This notion is problematic for Toering et al.’s (2012) dispositional SRL-SRS survey. Furthermore, if any survey were to be relevant to practitioners it would need to inform strategies that could be cultivated to directly enhance approaches in sport practice. Thus, a new line of research emerged; it drew from Toering et al.’s (2012) SRL-SRS but examined the value of self-reported SRL specifically in the context of sport practice. This aligned with recommendations that survey items represent the situational-specific demands of the behavioral context (Feltz and Chase, 1998). This pivot was predicated on the idea that SRL-SRS items originally intended for classroom-based SRL were inadequate for sport practice situations and that further testing and refinements were required to develop a more contextually-suited survey.

## Developing the construct validity of the self-regulated sport practice survey

This section traces the evidentiary development of a contextually-suited survey, the SRSP, across three investigations: Bartulovic et al. (2017), McCardle et al. (2018) and Wilson et al. (2021b). The result of this further testing is the SRSP survey (Supplementary Appendix A). See Supplementary Appendix B for an overview of information attesting to the construct validity of the SRSP. It is important to note that each of these serial investigations featured most of the current authors, thus there was continuity of inquiry in pursuit of a valid and reliable survey tool. Although many of the same investigators engaged continuously in the development of the *SRSP* survey, we recognize that readers often find the use of different acronyms for earlier versions of this survey awkward. For clarity, the evolution of the surveys occurred in this order, as outlined below: (1) the *SRL-SRS for Sport Practice* (Bartulovic et al., 2017); (2) the Self-Regulated Learning scale – Sport Practice, or *SRL-SP* (McCardle et al., 2018); (3) the *Self-Regulation of Sport Practice Survey*, or the *SRSP* survey (Wilson et al., 2021b).

Bartulovic et al.’s (2017) study was the initial effort to import Toering et al.’s (2012) *SRL-SRS* and make refinements so that it better suited the context of sport practice. A systematic vetting process of the SRL-SRS with external scholars versed in SRL and sport precipitated several refinements including (a) removing phrasing that was the vestige of questions about mathematics/science and replacing it with “during practice” and “practice tasks”, (b) making minor changes to ensure understanding by athletes, and (c) inserting a new preface prompting respondents to reflect on tasks done before, during and after sport practice. The result was the *SRL-SRS for Sport Practice* (Bartulovic et al., 2017). In a sample of 272 North American competitive athletes (ages 18–35; 200 men, 72 women), investigators used exploratory factor analysis to establish good measurement model fit; they reported a six-factor model that aligned with the same factors on Toering et al.’s (2012) SRL-SRS. The survey remedied some prior multicollinearity concerns among subscales, improved convergent validity, with reasonable divergent validity. It showed *criterion validity* in that survey scores representing greater overall engagement in SRL were more associated with being in an elite group, compared to less-elite and recreationally competitive groups. Further, scores for four SRL subscales were able to individually predict membership in more elite groups compared to less-elite groups. Bartulovic et al.’s (2017) survey thus demonstrated substantially improved *face validity* by specifically assessing psychological processes in relation to sport practice and showed promising evidence of criterion validity.

Next, McCardle et al. (2018) studied whether any conceptual or psychometric gaps had been created by importing the SRL-SRS to sport practice. They devoted attention to psychometrics with aims of ensuring fulsome conceptual representation of SRL processes (by testing new items and revisiting items from the original SRL-SRS) and coherent factorial validity. With 482 athletes (*M* age = 26.45, *SD* = 12.66; 265 women, 217 men), they tested different permutations of the SRL-SRS for Sport Practice using exploratory structural equation modeling. Findings confirmed a five-factor model with good fit, while balancing representation of broad SRL processes with parsimonious considerations for assessment. Prior issues with cross-loaded items between “evaluating” and “reflecting” were resolved with an integrated subscale (effectively reducing the survey from six to five subscales) and problematic self-monitoring items were trimmed to retain a “checking” subscale. They tendered a refined survey: the Self-Regulated Learning scale – Sport Practice, or *SRL-SP*.

McCardle et al. (2018) explained that a degree of inter-factor multicollinearity was acceptable because self-reports for these psychological subscales “are intertwined and enacted proximally to one another. It may be unrealistic to develop an athlete survey tool … which separates these subprocesses as distinct measurable factors at a level that satisfies strict psychometric criteria” (p. 8). They contended that the survey’s validity could not be judged on factorial validity alone and required equal consideration of criterion validity. To wit, they argued for fuller consideration of tests of group discrimination, citing these tests as a common mechanism for validating an expert advantage in sport expertise research. With such tests, the investigators showed how the subscale scores from the SRL-SP reliably discriminated between skill groups ranging from local to international levels, with small-to-medium effect sizes. “Evaluating-reflecting,” “effort,” and “self-efficacy for challenge” scores distinguished between the groups, with a particular advantage attributed to international-level athletes. Altogether, this study confirmed the conceptual rigor of the SRL-SP and advanced this survey as having a good combination of factorial and criterion validity.

**FIGURE 1:**
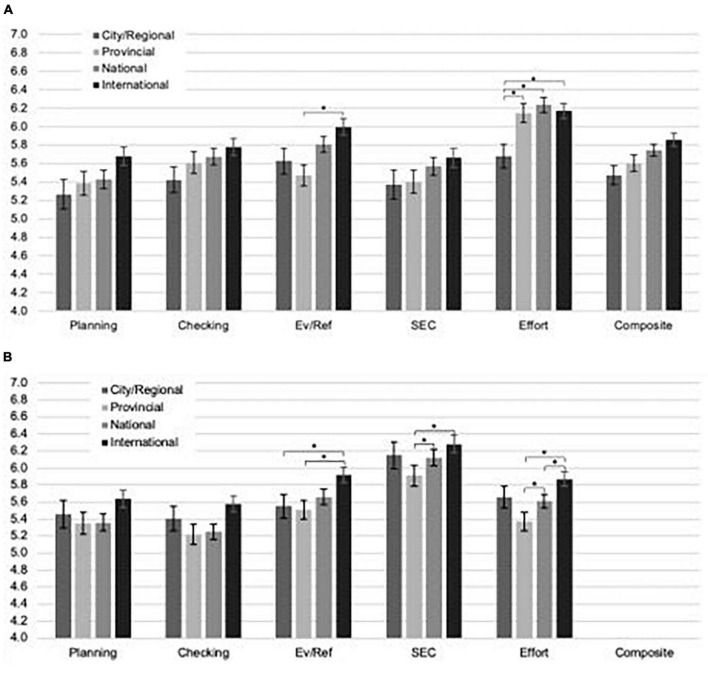
**(A)** Subscale and composite scores on the SRSP reported in Wilson et al. (2021b), presented by skill level group. **(B)** Subscale and composite scores on the SRL-SP reported by McCardle et al. (2018), presented by skill level group. *p < 0.05; Error bars represent standard error of the mean.

One more step followed that aligned the survey’s development with the principle of situational specificity. Wilson et al. (2021b) assessed how differences in SRL scores between skill groups (i.e., criterion validity) would be impacted by three methodological modifications: (a) an added preface that focused respondents on challenging sport tasks because situational challenge is a catalyst for SRL enactment (Hadwin et al., 2011); (b) insertion of five non-scored, reverse-coded items to prevent response bias; and (c) aligning all Likert-scale responses on agreement rather than frequency of SRL use. A sample of 235 North American, mixed-sport athletes (ages 13–42; 66.8% women, 32.8% men, 0.4% non-binary) completed McCardle et al.’s (2018) SRL-SP survey, with these modifications. Confirmatory factor analysis replicated the five factors of the SRL-SP with good fit, there was favorable internal consistency reliability, and no model refinements were required. Notably, Wilson et al. (2021b) focused on tests of criterion validity, with results showing enhanced effect sizes over McCardle et al. (2018), when comparing four skill groups (see Figures 1A, B). An omnibus MANOVA showed a small-to-medium effect, as did “planning” scores, with “effort” and “evaluating-reflecting” indicating medium effects. They interpreted that group discrimination was enhanced because high-level athletes more strongly reported metacognitive strategies when they were primed to think about goal frustrations and plateaus (i.e., challenge) around practice in the survey preface.

**FIGURE 2:**
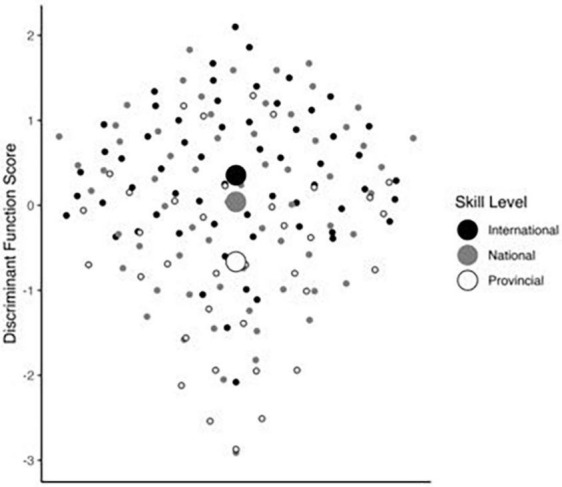
Discriminant function analysis (previously unreported) performed on data from Wilson et al. (2021b), in a sample restricted to high performance athletes ranging from provincial to international level. Small points represent discriminant function scores (i.e., the linear combination of relevant SRL processes) for each participant, varying on the y-axis and visually distributed on the x-axis. Large points represent group centroids for each of the skill level groups.

A discriminant function analysis (DFA) portrays such group discrimination visually for the provincial, national and international groups (see Figure 2). A DFA reports which combination of subscale scores best predicts group membership. After restricting the Wilson et al. (2021b) sample to provincial-level athletes or higher (*n* = 164, *M* age = 26.8, *SD* = 16.9; 114 women, 50 men), Figure 2 shows the resulting linear combination of SRL variables predicting skill group membership. One significant function was extracted (Eigenvalue = 0.15, Canonical Correlation = 0.36, *p* = 0.004), explaining 87.8% variance. Higher skilled membership was strongly influenced by higher scores on “evaluating-reflecting” (standardized coefficient = 1.22), moderately influenced by higher “self-efficacy for challenge” scores (stand coeff = 0.46), and weakly influenced by lower “planning” (stand coeff = −0.28). In this case, greater evaluating and reflecting and greater personal efficacy and coping during challenging practice scenarios were together predicting more elite status, with the possibility that planning was curtailed among elites. “Effort” and “checking” did not significantly improve group prediction in this case.

In light of the modifications they made to McCardle et al.’s (2018) survey, Wilson et al. (2021b) titled this recent survey the SRSP. Results from the SRSP offer strong evidence in terms of reliability and validity of assessing SRL in relation to sport practice. The enhanced factorial and criterion validity of this survey are a product of the cumulative efforts preceding it. Furthermore, the utility of such a sport-practice self-regulated survey is gaining acknowledgment beyond the initial clusters of researchers. For instance, Reverberi et al. (2021) tested an Italian version of the SRL-SRS for Sport Practice and found that the scores performed well in a sample of professional, semi-professional and amateur soccer players.

## Where’s the practical validity in all of this?

Many avenues exist to establish validity in the social sciences (Drost, 2011); however, perhaps the least considered by researchers is practical validity. Practical validity refers to the fulsome consideration of how research findings/products, in our case the SRSP, are informed by the perspectives of practitioners, located within the narratives of applied practice, and received instrumentally by those in practice. The SRSP was never intended to be a diagnostic test dedicated to measurement alone; its development was pursued such that it could be informed by, and could inform, discussions in applied practice. Throughout the survey development process, we have been aware of concerns that “theoretical concepts are often not tested in applied settings … and this lack of integration between applied work and theoretical research … has created a disconnect between practice and research in sport psychology” (Kontos and Feltz, 2008, p. 11). Consequently, we ensured that SPCs were involved in the external vetting of items for Bartulovic et al.’s (2017) survey and were central in discussing applied considerations in relation to a short form version of the SRSP, now in development (Wilson et al., 2019). However, we submit that it is time to examine the use of the SRSP more broadly among practitioners, particularly consultants who work on the psychology of practice, to gauge its utility and worth in the field, and determine any refinements or resources that will need to accompany its use.

Outside of pilot work with applied consultants, initial insights into how the SRSP could be used in an applied context came in a Polish case study. Siekańska et al. (2020) validated a short-form version of the SRL-SP in Polish and had an experienced SPC implement it in her discussions with a highly elite free-diver. The athlete first completed the survey in reference to his current training experiences, and then reflected on how he may have responded as a sub-elite athlete six years earlier, before he implemented mental training into his practice. The SPC explored his response reasoning and how each item pertained to his quality practice efforts. The athlete’s consistently high current scores were reflected in discussion as he explained his highly introspective and analytic approach to self-training. Discussion about how his practice approaches had changed over time focused on five survey items. He had learned to better plan and prioritize core components before starting practice, yet during the practice tasks, he had grown more comfortable transitioning from attending to technical steps to letting them happen automatically. Post-practice, it had become easier for him to evaluate his weaknesses because he also learned to simultaneously seek information to evaluate strengths, which maintained his self-confidence. Siekańska et al. (2020) concluded that the survey could inform fruitful discussion on how athletes manage their own learning via planning, monitoring, and corrective methods in training. They saw it as a useful dialogue tool for helping athletes develop proactive SRL approaches to their practice tasks. As this athlete was an admitted “self-learner and explorer” and engaged in substantial self-coaching in a very unique sport, they commented that the applicability of the survey might prove specific to a sport discipline and/or an athlete’s competitive level and might depend on the professional support/coaching guidance ascribed to the athlete’s context.

### What is the scope of self-organized practice?

As we have introduced sport managers, coaches, and SPCs to the SRSP, some have asked, “how much of an opportunity is there really for elite-level athletes to manage aspects of their training?”. In an amateur sport system in which increasing professionalization imposes structures/constraints (e.g., coach-prescribed activity, centralized regimes) on what athletes do, it is fair to query whether this means that athletes have less latitude to dictate what they manage or decide to do around practice.

There is recent research that explored this question. Bain et al. (2020) conducted survey research examining the proportion of time athletes spent in self- vs. coach-organized practice. Canadian athletes (*N* = 226; 66% women, 34% men) from 15 to 42 years of age (*M* = 23.1), from individual (61.2%) and team (38.8%) sports, recruited broadly from sport clubs/organizations and national sport centers, completed the SRSP and questions about amounts of structured sport practice in the past week. These athletes, who ranged from city to international levels, also judged the percentage of time they were in self-organized practice activities at that point in the season, with 100% representing “all my time is spent in activities I have organized, designed or initiated.” They were asked to:

*Think of only those activities you have organized for yourself, designed or initiated on your own, even if other athletes/players joined you. Think of activities where a coach was not present. *Self-organized practice* can take many forms, including activities you planned and structured for yourself, skill/technique work, playful games, conditioning work, mental rehearsal/sport psychology practice.*

Descriptive statistics, ANOVA and correlations explored how athletes’ responses for self-organized practice (SOP) related to sport type, phase of season, skill level, years of training, and SRSP scores.

**FIGURE 3:**
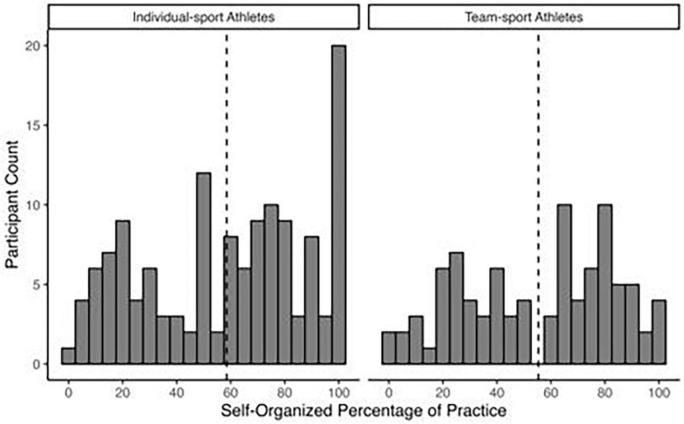
Distributions of perceived self-organized practice time from 0 to 100% of total sport training for individual-sport athletes **(left)** and team-sport **(right)** athletes, from Bain et al. (2020). Each bar represents a bin of 5 percentage points in width. Dotted lines indicate the mean percentage of perceived self-organized practice time by sport type.

**FIGURE 4:**
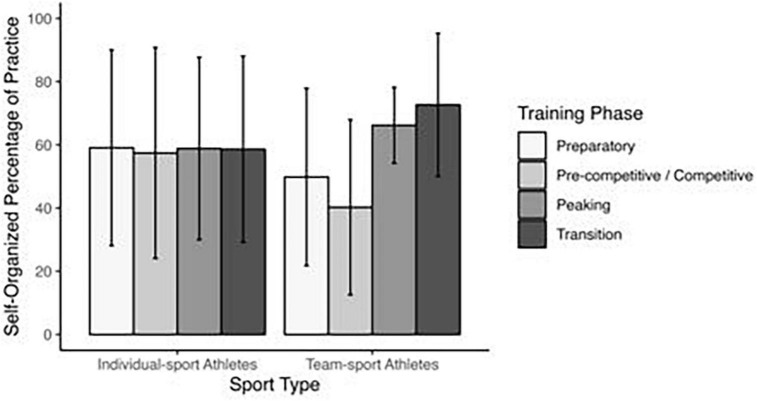
Means of percentages of self-organized sport practice as a function of athletes’ sport type and phase of season, from Bain et al. (2020). Error bars represent standard deviation.

The sample had SOP representing over half of practice time (*M* = 57.2 ± 29.7%). Distributions were neither normal nor positively skewed as might be expected (see Figure 3), warranting further exploratory analyses. A two-way ANOVA for sport type (individual/team) by phase of season (i.e., preparatory, pre-competition/competition, peaking, transition) showed no main effect for sport type (*p* = 0.43), but the interaction was significant, *F*(3, 217) = 2.99, *p* = 0.03. In the pre-competition/competition phase, individual sport athletes had a higher percentage than team sport athletes, *p* = 0.04 (see Figure 4). Individual sport athletes’ SOP was stable across all phases (*p* = 0.99) whereas team athletes had higher SOP in transition (e.g., out-of-season training) than preparatory and pre-competition/competition phases, both *p*s < 0.01 (Figure 4).

A one-way ANOVA for skill group differences (i.e., city/regional, provincial, national, international) in SOP for athletes competing at the senior (18+) age group was non-significant, *p* = 0.65. Yet, years of training and SOP were correlated, *r*(224) = 0.285, *p* < 0.01. To unpack this further, Bain et al. (2020) conducted a tertile-split on athletes’ data for years of training. A one-way ANOVA using these tertile groups was significant, *F*(2, 219) = 10.60, *p* < 0.01. Tertile splits were equal to 5 (33^∧^) and 10 years of training (66^∧^), creating groups of high (*n* = 60; *M* training years = 14.9, *SD* = 3.4), medium (*n* = 89; *M* training years = 7.9, *SD* = 1.5) and low training experience (*n* = 73; *M* years = 3.8, *SD* = 1.2). The high training group reported greater SOP (*M* = 70.8 %, *SD* = 27.2) than the medium (*M* = 55.9 %, *SD* = 26.9) and low group (*M* = 48.15%, *SD* = 31.22) (both *ps* < 0.01), with the medium no different from the low group, *p* = 0.08.

The final analyses tested associations between SOP and reported use of SRL processes, using composite and subscale scores from the SRSP. “Planning” correlated with SOP, *r*(224) = 0.15, *p* = 0.02, but all other correlations were *p* > 0.21. When inspecting only the most elite group alone—athletes on senior international teams (*n* = 62; *M* SOP = 54.9 %, SD = 32.52; 37 and 25 from individual and team sports, respectively), “planning” (*r* = 0.27, *p* = 0.03) and “evaluating-reflecting” (*r* = 0.28, *p* = 0.02) associated with greater SOP. All other correlations were *p* > 0.12.

In sum, Bain et al. (2020) showed there are contextual circumstances implicated in the scope of SOP time. As SOP time is not supervised by a coach, its utility for skill acquisition may depend substantially on how athletes engage in leading their own design, initiative, organization, and evaluation of practice activity. Future narratives on SRL and the SRSP might determine opportunities for “value-added to practice” with a lens on athlete self-supervision and self-management. Moreover, in the vein of translating research to practice, we believe an understanding of contextual variables is imperative for beginning to locate the SRSP within consulting praxis. Bain et al.’s (2020) results demonstrate that SOP time will vary in importance based on the phase of the season in team sports but is more stable in individual sports. Researchers have yet to decipher a skill-group difference for perceptions of SOP, but the amount of self-managed activity appears to be, in part, related to years of experience and perhaps maturity in sport training. Although some might challenge the reliability of a single-item SOP measure, the question was clear and had face validity. The responses indicate plainly that many athletes believe they have ownership of large portions of self-organized time.

Bain et al.’s (2020) reports are aligned, for example, with Young’s (1998) study of Canadian middle-distance runners, which showed that elite 15–19 year-olds spent more than 80% of their training not supervised by a coach. The implication is that SRL matters for self-organization, especially in an unsupervised context. Being able to verify/assess the quality of one’s own planning, selecting information, detecting and correcting mistakes, are crucial characteristics of self-regulated learners. Even when a coach is present, such as in traditional team sports, there is room for athletes to experiment technically to employ self-regulated competencies and enact their agency for skill acquisition efforts, aside from direction/prescription from a coach. In sports like figure skating and swimming, coaches look favorably on athletes who develop such competencies because they help to optimize practice time and enrich athlete-coach dialogue (Bain et al., 2021). Athletes are responsible for engaging in cycles of personal assessment and self-generated feedback about their practice performances to communicate with their coach to help make strategic adjustments. Altogether, we see these contexts as representing possible “margins of gain” in high performance sport, justifying a focus on the psychology of practice that features the SRSP.

### How can practitioners begin to think about using the SRSP?

The SRSP could be situated within consulting to facilitate dialogue about metacognitions and motivations around practice. We are aware of the longstanding credulous-skeptical debate around testing in applied sport psychology, and the hesitancy of some consultants to use survey tests because of negative connotations around psychometrics, clinical diagnostics, or because many instruments pass the research litmus test but feel disjointed from real-world practice (Marchant, 2010). The aforementioned review of the evolution of the SRSP underscores the integrity of the survey for assessment purposes in the research domain; however, we are uncomfortable referring to the SRSP as a “test.” The intention behind asking a client to complete the SRSP is to generate feedback and content for consultation and thus it is important to scrutinize how to locate the SRSP as a reliable and valid tool for enhancing dyadic dialogue around the psychology of practice. The SRSP is one tool that could enrich dialogue on the psychology of practice, but it ideally should be merged with interviewing (i.e., survey as an interview tool) as an “exercise providing insight into how well athletes know themselves” (Silva et al., 2011, p. 105), and corroborated by observation.

In service of the applied side of sport psychology, the SRSP provides athletes and practitioners with a language around the skills of learning how to learn. “Learning” is translated from an abstract construct to a set of concrete thoughts, actions, and behaviors. This, in turn, can facilitate rich discussions about the quality of athletes’ SRL because of a shared vernacular, that can be specifically applied to encourage athletes to introspect on quality sport training.

It is our hope that the SRSP can be helpful in counseling athletes to transition from thinking about “being a better learner” to “how to engage in better learning.” The SRSP has the potential for creating self-awareness, heightening athletes’ beliefs that they can engage in their own learning, situated in discussions about how they can evaluate and learn from their own metacognitions without always relying on a coach. A practitioner could ask an athlete to consider their interpretation of their scores on the SRSP, asking them to reflect on what the scores mean to them, and guiding them to place the scores and meanings within the context of optimizing SRL. A practitioner could ask an athlete to ponder how someone who is more elite in their sport might score themselves. Probing how they see their own SRL initiatives, how their processes compare to where they wish to be, and what that latter aspiration might look like for them—these may all be “meaning making” exercises that follow from using the SRSP in dyadic consultancy work. These exercises could create greater insight around optimal practice behaviors and permit the athlete to explore and adapt in relation to different behaviors. Identified gaps between the current self and the athlete’s projection to an elite self might bring attention to how hard work is enacted, inviting a growth mindset (Dweck, 2016), and bringing meaning and self-accounting to being a matured “student of one’s sport practice.”

The SRSP offers athletes metrics (in a personal but not an absolute sense) that could be used over time to gain insight into SRL skill usage and improvement. Generally, the numerical scales represent the extent to which an athlete agrees with the use of SRL processes; still, SPCs cannot assume that higher agreement on use is equivalent to “better” self-regulated practice. An athlete can apply their own interpretation to a score to determine the range/nature of SRL skill use that is associated with optimized learning in *their* self-directed training sessions. The emphasis on “I” in the wording of the SRSP items invites athletes to attend to self-agentic narratives, rather than attending to socially comparative or normative considerations.

Our own pilot work with SPCs suggests that the SRSP could feature in discussions that encourage an athlete to define what it means to be a reflective learner, to be evaluative, and to introspect on the criteria they use to self-appraise as well as the valence (overly positive or negative) nature of their evaluation-reflection. The SRSP offers sufficient breadth in various subscales, whereby SPCs can work with athletes to identify areas of attention or remediation. What this looks like might depend on how athletes and practitioners use the survey in tandem, and in relation to preferential approaches of the consultant and personal qualities and individual difference characteristics of athletes (Piepiora, 2021; Piepiora et al., 2022). Research could interrogate best practices for how SPCs support athletes as they make meaning of their SRSP scores and “step” past their scores to elaborate and refine strategies. In concert with this, there are three key questions: “how do SPCs see the potential utility of a survey for self-regulated sport practice for their clients and in their consulting services?”; “how might SPCs wish to design a narrative around the SRSP to go about influencing athletes’ scores?”; and “what types of impediments prevent SPCs from using it to influence athletes’ scores?”.

Research on SRL from multiple domains suggests that SRL skills are dynamic, change over time, and are developmental attributes that athletes can learn to modulate to their advantage. The goal in developing the SRSP was to eventually consider its integration into narratives in sport psychology interventions about optimizing deliberate practice. The SRSP might also be useful for monitoring changes in the use of SRL skills as a result of consultancy interventions. For the SRSP to be truly tested in terms of practical validity, attention should now turn to understanding how the community of practitioners sees and communicates its use, whether SPCs wish to act upon it with their expertise, and the resources they believe need accompany it. Future inquiry could examine resources/conditions that facilitate its use and restrictions/barriers to its use. To this end, we contend that there is a need for practitioner reflection and evaluation, followed by any necessary adaptation to facets of the SRSP and its emerging praxis (e.g., supporting curriculum, guidelines). Indeed, the accumulation of such procedural knowledge is essential to gain the confidence of SPCs in validly advancing the translation of the theoretically-informed SRSP into dialogue on the psychology of practice.

## Data availability statement

The datasets presented in this article are not readily available due to privacy/ethical restrictions. Data are available from the University of Ottawa Office of Research Ethics and Integrity (ethics@uottawa.ca) for researchers who meet the criteria for access to confidential data. Requests to access the datasets should be directed to BY, byoung@uottawa.ca.

## Ethics statement

The studies involving human participants were reviewed and approved by the University of Ottawa Office of Research Ethics and Integrity H-08-21-7176. Written informed consent to participate in this study was provided by the participants’ legal guardian/next of kin.

## Author contributions

BY was the primary writer, the primary supervisor of all materials, works and personnel on works informing this manuscript, and principal grant holder on all supporting works. SW was involved in secondary interpretations of writing on this manuscript, led a prior research work that informed parts of this manuscript, and a co-investigator on original data with LB. SH provided secondary interpretations poignantly related to applied and consultancy perspectives on this manuscript and involved in pilot interviews that informed insights into consultancy using the survey featured in this manuscript. LB led data collection resulting in the original data analyses on the scope of self-organized practice. MS led data collection and interpretation of a qualitative case study that featured the survey in this manuscript employed in consultancy practice. JB was a co-investigator on the vast majority of works informing this manuscript, co-investigative grant holder on most of these works, and provided interpretive and editing support on the writing of this manuscript. All authors contributed to the article and approved the submitted version.
