# Supplementary material for: On the self-regulation of sport practice: Moving the narrative from theory and assessment toward practice
Source: Front Psychol. 2023 Mar 28;14:1089110. doi: 10.3389/fpsyg.2023.1089110 (PMC10086193; doi:10.3389/fpsyg.2023.1089110)
Supplement: Supplementary file 1 [file Table_1.pdf]

Appendix A

The Self-Regulation of Sport Practice (SRSP) Survey

Preface

Think about your most important practice sessions each week. Within these sessions you engage in many tasks and not all practice segments are equally relevant to your development as an athlete. We would like you to think in particular about those practice tasks that challenge you. For example, they may challenge you because they address your specific performance gaps between where you are now and where you want to be. They may demand a lot of mental and physical effort. Because these important tasks target the areas that you have to work on the most, they might not be enjoyable but they are important for your development.

Please read the following statements and choose the response that best describes you. There are no right answers – please describe yourself as you are, not how you want to be or think you should be.

Strongly Disagree	Disagree	Slightly Disagree	Neither Agree or Disagree	Slightly Agree	Agree	Strongly Agree
1	2	3	4	5	6	7

1. I determine how to approach a practice task before I begin.	1	2	3	4	5	6	7
2. I do not check how well I am doing during a practice session.	1	2	3	4	5	6	7
3. After finishing, I look back on practice tasks to evaluate my performance.	1	2	3	4	5	6	7
4. I concentrate fully when I do a task at practice.	1	2	3	4	5	6	7
5. When facing difficulties at practice I can rely on my coping abilities.	1	2	3	4	5	6	7
6. Before practice tasks, I carefully plan my course of action.	1	2	3	4	5	6	7
7. Before practice tasks, I consider the parts of the task I have to complete.	1	2	3	4	5	6	7
8. I do not set specific goals before starting practice tasks.	1	2	3	4	5	6	7
9. I check aspects of my workout while doing it.	1	2	3	4	5	6	7
10. I think about my past experiences at practice to gain new insights.	1	2	3	4	5	6	7
11. I usually put forth my best effort when performing tasks at practice.	1	2	3	4	5	6	7
12. I am confident that I can deal efficiently with unexpected events at practice.	1	2	3	4	5	6	7
13. I try to understand the goal of a practice task before I do it.	1	2	3	4	5	6	7
14. Before practice tasks, I figure out what I need to do to accomplish my goals.	1	2	3	4	5	6	7
15. I reflect upon my actions at practice to see whether I can improve them.	1	2	3	4	5	6	7
16. I am unable to handle unanticipated challenges during practice.	1	2	3	4	5	6	7

17. I usually keep working hard even when sport training tasks become difficult. 1 2 3 4 5 6 7
18. When I am confronted with a difficulty during practice, I can usually find several solutions. 1 2 3 4 5 6 7
19. I think about what a practice task requires me to do before I do it. 1 2 3 4 5 6 7
20. Before I do a practice task, I think through the steps in my mind. 1 2 3 4 5 6 7
21. I look back to check if what I did in practice was right. 1 2 3 4 5 6 7
22. When thinking about my practice, I reflect about my strengths and weaknesses. 1 2 3 4 5 6 7
23. I know how to handle unforeseen situations during practice, because I am resourceful. 1 2 3 4 5 6 7
24. I have trouble maintaining my best effort when practice tasks become difficult. 1 2 3 4 5 6 7
25. I don't give up at practice even if a task is hard. 1 2 3 4 5 6 7
26. I clearly plan my course of action before starting practice tasks. 1 2 3 4 5 6 7
27. I look back to see if I did the correct procedures at practice. 1 2 3 4 5 6 7
28. I do not evaluate whether I am getting better from practice to practice. 1 2 3 4 5 6 7
29. Before practice tasks, I figure out my goals. 1 2 3 4 5 6 7
30. No matter what comes my way at practice, I am usually able to handle it. 1 2 3 4 5 6 7
31. Even when I don't like a task during practice, I work hard. 1 2 3 4 5 6 7

Note. In an online platform, the preface should be at the top of the first page. If items are spread across multiple “pages”, all subsequent pages should include the following instruction at the top of the page: “Remember to keep in mind the types of practice tasks that really challenge you during important training sessions.”

SCORING:

To get a score for each subscale, average the following items:

Planning	1, 6, 7, 13, 14, 19, 20, 26, 29
Checking	9, 21, 27
Evaluating/Reflecting	3, 10, 15, 22
Self-Efficacy for Challenge	5, 12, 18, 23, 30
Effort	4, 11, 17, 25, 31

The following, negatively-worded items are included to protect against response bias. They are NOT to be scored as part of any subscale:

Negatively-worded items	2, 8, 16, 24, 28

Appendix B

Evidence for the Construct Validity of the Self-Regulated Sport Practice Survey

Content Validity: ensuring that the survey items are defined theoretically, that they thoroughly cover the theoretical domain and relevant dimensions, and that the theoretical dimensions are accepted by peers	• Young and Medic (2008) advocated for Zimmerman's (1998) model of self-regulated learning to be considered as the underpinning for understanding optimal sport practice (also see McCardle et al., 2019)<br>• Toering et al.'s (2009, 2012) work on the SRL-SRS survey, the predecessor to the SRSP, defined self-regulated learning according to theoretically valid frameworks: Zimmerman and Schunk's (2001) social-cognitive model and Ertmer and Newby's (1996) model of a reflective learner. They ensured the survey subscales drawn from the education domain accurately represented theoretical dimensions of self-regulated learning.<br>• Toering et al. (2011): Six expert coaches identified behaviours characterizing self-regulated learning in soccer players. These behaviours were observed during sport practice and were associated with SRL-SRS survey scores.<br>• McCardle, Young, and Baker (2018) revisited theoretically relevant items discarded by Toering et al. (2012) and Bartulovic, Young, and Baker (2017) and explored the fit of new items, ensuring that survey dimensions comprehensively considered the theoretical scope of self-regulated learning and that they had been submitted to measurement models for factorial validity.
Face Validity: ensuring that constructs are accurately translated into operationalization, based on subjective judgment	• Bartulovic, Young, and Baker (2017): Nine academic experts in the domain of self-regulated learning and sport/motor learning, independent from the researchers, rated the survey items to ensure they fit with the definitions of their respective subscale, and whether they would generate data about how athletes are active participants in their own learning. This resulted in key refinements to the survey preface and wording of items to focus on tasks done before, during and after sport practice.

Factorial Validity: ensuring a survey is measuring what it is intended to, using factor analysis. Factor analysis determines the degree to which a specified underlying structure of a scale is recoverable in a sample of survey scores.	• Bartulovic, Young, and Baker (2017) used exploratory factor analysis with principal factor analysis (direct obliminal rotation) to arrive at a 6-factor, 31-item model.	Fit indices: CFI = .90, SRMR = .078, IFI = .90, RMSEA = .052 (90% CI .045- .058), χ^2/df = 1.72<br><br>Factor loadings: planning (.61-.84); self-monitoring (.44-.75); evaluation (.71-.81); reflection (.50-.78); effort (.57-.81); self-efficacy (.70-.80).<br><br>Convergent validity of subscales: 4 subscales had average variance explained > .50.<br><br>Discriminant validity of subscales: all subscale correlations: < .68, except "planning" and "evaluation" (.76). No factor cross-loadings were within .20 absolute value of primary factor loadings. Three subscales had values for maximum share variance < average variance explained.<br><br>Cronbach values: .62-.87
	• McCardle, Young, and Baker (2018) used exploratory structural equation modeling to reject a theoretically extended measurement model with reclaimed and new items. Using exploratory structural equation modeling on a calibration sample, followed by confirmatory factor analysis (robust maximum likelihood estimation) for the resultant measurement model on an independent sample, they confirmed a 5-factor, 26-item model.	Fit indices: CFI = .91, TLI = .91, RMSEA = .060 (90% CI .052- .068)<br><br>Factor loadings: planning (.67-.85); checking (.74-.81); evaluation-reflection (.64-.78); effort (.64-.87); self-efficacy for challenges (.71-.78).<br><br>Convergent validity of subscales: All average variance explained values were > .50.<br><br>Discriminant validity of subscales: all subscale correlations were < .71, except "planning" and "evaluation-reflection" (.87,), "checking" and "evaluation-reflection" (.86). Maximum shared variance values were .36-.76. Average shared variance values were .20-.51.<br><br>Composite reliability scores > .70 for all subscales.

	• Wilson et al. (2021) performed a confirmatory factor analysis (robust maximum likelihood estimation) in a new sample on the resultant model from McCardle, Young, and Baker (2018). There were 5 reverse-scored items to prevent response bias (i.e., the final 31-item SRSP survey), but these items were not intended for factor analysis.	Fit indices: CFI = .92, TLI = .91, RMSEA = .047 (90% CI .038- .056)<br><br>Factor loadings: planning (.53-.79); checking (.48-.75); evaluation-reflection (.52-.70); effort (.53-.84); self-efficacy for challenges (.63-.76).<br><br>Subscale correlations: < .75, except "checking" and "evaluation-reflection" (.95).<br><br>Cronbach values: .64-.88; MacDonald's Omega values: .69-.89
Criterion Validity: ensuring correspondence between the survey's measures and established external referents. The ability of a construct to validly predict skill group membership within a mixed-skill sample is critical.	• Bartulovic, Young, and Baker (2017): Overall SRL scores predicted being in an elite group compared to a less-elite and recreationally competitive group. In analyses with all subscales simultaneously, self-monitoring predicted membership in the elite and less-elite group compared to the recreationally competitive group. Separate subscale analyses showed "planning", "self-monitoring", "effort and "self-efficacy" predicted membership in the elite group.	Effect sizes in multinomial logistic regressions for overall SRL scores:<br><br>3 groups: Nagelkerke R^2 = .30; odds ratio for elite group was 3.15 (classification rate was 43% better than by chance) with recreational competitive group as referent.<br><br>2 groups: Nagelkerke R^2 = .09; odds ratio for elite group was 1.82 (classification rate was 20% better than by chance) with less-elite group as referent.<br><br>Effect sizes in multinomial logistic regressions for all SRL subscale scores simultaneously:<br><br>3 groups: Nagelkerke R^2 = .35; odds ratio attributed to "self-monitoring" for elite group was 2.75 (classification rate was 46% better than by chance) and less-elite group was 2.25 (8% better), with recreational competitive group as referent.

	• McCardle, Young, and Baker (2018) conducted a MANOVA comparing 4 groups (local, provincial, national; international) on SRL subscale scores. The scores generally distinguished international level athletes from less-skilled groups. A follow-up discriminant function analysis discriminated the international group from the rest, with the largest difference between international and provincial athletes. This function affirmed the contribution of "evaluation-reflection", "effort"', and "self-efficacy for challenges", but also showed smaller contributive roles for "planning" and "checking".	Effect sizes in MANOVA: Omnibus Wilk's λ = .91, F = 2.07, *partial* η^2 = .029 (small).<br>Univariate ANOVAs by subscale:<br>"Evaluation-reflection" (*partial* η^2 = .032; small): international > provincial and international > local.<br>"Effort" (*partial* η^2 = .043; small): international > provincial and national > provincial.<br>"Self-efficacy for challenges" (*partial* η^2 = .051; small-to-medium): international > national, international > provincial, and national > provincial.
	• Wilson et al. (2021) contended that descriptive statistics for "planning", "effort" and "self-efficacy for challenges", and overall SRL, showed complete correspondence (i.e., mean SRL scores escalated in step with skill group status). They conducted a MANOVA comparing 4 skill groups (city, provincial, national; international) on SRL subscale scores, with results showing stronger effect sizes for criterion validity than in McCardle, Young, and Baker (2018). Controlling for the effects of social desirability of self-report, showed that results did not change meaningfully.	Effect sizes in MANOVA: Omnibus Wilk's λ = .82, F = 2.55, *partial* η^2 = .064 (medium).<br>Univariate ANOVAs by subscale:<br>"Evaluation-reflection" (*partial* η^2 = .072; medium): international > provincial.<br>"Effort" (*partial* η^2 = .070; medium): international > city, national > city, and provincial > city.
External Validity (Practical Validity): ensuring the conclusions of scientific study apply outside the context of the study, including that the implications translate validly from theory to practice. This may involve specifying	• In developing a short form of the SRSP survey, Wilson et al. (2019) had a professional sport psychology consultant vet the survey items for their relevance to applied practice.<br>• Siekańska et al. (2020) had an experienced sport psychology consultant implement the survey in her discussions with a highly elite athlete. They concluded that the survey was a useful dialogue tool for helping athletes develop proactive SRL approaches to their practice tasks.	

circumstances in which the conclusions generalize across situations, people, and time.	• Bain et al. (2021) had an experienced high-performance coach discuss the utility of the survey and how he would use it to engage with his athletes and the conditions under which he would use it. They concluded that the coach saw great value in using the survey to guide conversations around learning and practice skills.	

Note. SRSP = Self-Regulation of Sport Practice Survey; SRL-SRS = Self-Regulated Learning – Self-Report Scale.
